# Ginsenoside-Rb1 Induces ARPE-19 Proliferation and Reduces VEGF Release

**DOI:** 10.5402/2011/184295

**Published:** 2012-01-04

**Authors:** Brandi S. Betts, Kalpana Parvathaneni, Bharat B. Yendluri, Jeffery Grigsby, Andrew T. C. Tsin

**Affiliations:** Department of Biology, The University of Texas at San Antonio, San Antonio, TX 78249, USA

## Abstract

Rb1, a ginsenoside from ginseng root extract, possesses antiangiogenic effects, but its role on ocular cells has not been studied. We hypothesize that Rb1 inhibits the production of the angiogenic cytokine VEGF from ARPE-19 cells, leading to a significant reduction in the proliferation of ocular vasculatures. Data from our experiments show that Rb1 induced an increase in the number of ARPE cells in culture, while VEGF release (pg/10,000 viable cells) was significantly reduced. Treatment with VEGF and cotreatment with Rb1 and VEGF showed that this Rb1-induced cell proliferation was mediated by VEGF. Because VEGF from RPE plays a major role in promoting angiogenesis in ocular vasculatures. Our finding that Rb1 inhibits the release of VEGF from RPE cells suggests that Rb1 has a significant role in the eye to protect against angiogenic diseases such as age-related macular degeneration.

## 1. Introduction

Ginseng,* Panax ginseng* is a popular herb with a long history of medicinal use. Chinese have used ginseng for more than a thousand years to improve energy and longevity. The genus name “*Panax*” was given by a Russian botanist C. A. Meyer and is derived from Greek words “pan” meaning all and “axos” meaning cure. The species name “*ginseng*” comes from the Chinese word “rensheng” which means “human” as ginseng roots resemble the human body [1]. In the United States, it ranks among the top five herbal products sold and is an ingredient in energy tonics and used as an immunostimulatory dietary supplement [2]. It is a potent inhibitor of angiogenesis *in vivo *and* in vitro*, however, the underlying mechanism remains unknown [3]. Ginsenosides (or ginseng saponins) represent the principle active ingredients in ginseng and more than thirty different analogues have been identified [4]. Ginsenosides, Rb1 and Rg1, are the two major active constituents of ginseng. Ginsenosides belong to a family of steroids and share structural characteristics with steroid hormones [3] such as a four-ring steroid-like structure with an attached sugar [5].

*Age-related macular degeneration (AMD)* is a progressive eye disease which is the most common cause of irreversible vision loss and blindness in people aged 60 or older. It is characterized by angiogenesis, the abnormal growth of new blood vessels [6], which leak fluid and blood into the retina, inducing scar formation and destroying central vision. AMD is usually defined as either “dry” or “wet” and is a slow progressive disease with genetic influences as well as environmental risk factors such as cigarette smoking and perhaps diet and lifetime light exposure [7].

*The retinal pigment epithelium (RPE)* sustains visual function of the eye as the major metabolic gate keeper between photoreceptors in the retina and the choriocapillaries. The RPE and Bruch's membrane (BM) suffer cumulative damage over lifetime, which is thought to induce AMD in susceptible individuals [8–10]. By virtue of its location, and presence of tight junctions, RPE cells facilitate selective molecular transport between the choroidal blood and the outer neural retina, thus forming the outer blood-retina barrier (BRB) which prevents the passage of large molecules from choriocapillaries into the retina [11]. In addition, the RPE is responsible for phagocytosis and degradation of shed photoreceptors' outer segments. The disruption of these processes has been shown to result in retinal degeneration in experimental animal models and defects in the RPE contribute to initiation and/or progression of AMD in humans [11]. Although the vision loss of AMD results from photoreceptor damage in the central retina, the initial pathogenesis involves degeneration of RPE [12, 13]. Central to photoreceptor survival and function, the RPE is the major source of angiogenic (e.g., vascular endothelial growth factor (VEGF)) and antiangiogenic (e.g., pigment epithelium-derived factor (PEDF)) factors and therefore plays a central role in the modulation and progression of choroidal neovascularization [14–16] leading to AMD. Therefore it is essential to understand the changes in RPE cell and their release of angiogenic cytokines to mediate the propagation of diseases such as AMD.

*Vascular endothelial growth factor (VEGF)* is a pro-angiogenic growth factor (cytokine) which contributes significantly to the pathologic neovascularization in ischemic ocular diseases such as diabetic retinopathy, persistent hyperplastic vitreous syndrome, retinopathy of prematurity and choroidal neovascularization. VEGF is the key signal used by oxygen-hungry cells to promote growth of blood vessels. VEGF is a survival factor for endothelial cells of vasculature both *in vitro* and *in vivo. *It binds to specialized receptors on the surfaces of endothelial cells and directs them to proliferate leading to new vessels [17]. Although endothelial cells are the primary target of VEGF, several studies have reported its mitogenic effects on nonendothelial cells [18]. Recent studies show that RPE barrier integrity is modulated by VEGF through apically oriented VEGF-R2 receptors and thus, there is a growing body of evidence that intraocular VEGF can increase the permeability of both the inner (as in capillary walls) and outer blood-retina barriers, contributing to the accumulation of sub-retinal fluid and macular edema [19]. VEGF-A is necessary for cell survival, but has been implicated as a key protein involved in diseases associated with angiogenesis [20].

In the present study, significant effects were observed when human ARPE-19 cells in culture were treated with different concentrations of ginsenoside Rb1. This is the first study to report Rb1-induced RPE cell proliferation and reduces VEGF release (per 10,000 viable cells). These novel findings extend the known antiangiogenic effect of ginsenosides to the ocular tissues. Extensive investigations into the role of Rb1 in the prevention of ocular angiogenesis could lead to new methods of prevention and treatment of vascular eye diseases such as AMD and diseases of the retina.

## 2. Material and Methods

*Rb1* is a chemically pure saponin which is the principle ingredient of ginseng (a gift from Dr. R. N. S. Wong, Hong Kong Baptist University). Half-life of Rb1 is 16.7 hours noted in rat studies by Qian and Cai [21].

### 2.1. Culture of Retinal Pigment Epithelial Cells

Adult retinal pigment epithelial cells (ARPE-19) were obtained from the American Type Culture Center (ATCC—catalog number: CRL-2302). Cells were grown to confluence in Dulbecco's Modified Eagle's Medium (DMEM) containing 5.5 mM glucose and 10% FBS at 37°C + 5% CO_2_. Cells were seeded into 24-well plates at a density of 20,000 per well and incubated for 24 hrs prior to serum starving and treatments.

### 2.2. Rb1-Induced RPE Proliferation: Time and Dose Dependency Experiments

ARPE-19 cells were cultured as described previously [14]. After serum starving for 24 hrs, cells were treated with serum free media (SFM) containing 0.0, 0.25, 2.5, 25, 250 nM Rb1, respectively, for 24, 48, 72 hrs. After 24, 48, and 72 hrs, the conditioned media was collected and stored at −20°C for cytokine determination. Cells were removed and counted using a Neubauer Hemacytometer and trypan-blue exclusion method per manufacturer's instructions.

### 2.3. Exogenous Treatment of VEGF on ARPE-19 Cells

ARPE-19 cells were cultured as described previously and treated for 24 hrs with SFM containing 0.006, 0.06, 0.6, and 6 pg/mL of VEGF (VEGF was a generous gift from Dr. Clyde Phelix of UT, San Antonio), respectively. Cells were removed and counted using cell counting methods described previously.

### 2.4. Exogenous Cotreatment of VEGF and Rb1

The effect of cotreatment on cell proliferation and cytokine release by ARPE-19 cells were carried out by co-treated cells with SFM containing 250 nM Rb1 and exogenous VEGF in concentrations described previously (0.006, 0.06, 0.6, and 6 pg/mL of VEGF, resp.). The conditioned media was collected and stored at −20°C for cytokine determination. Cells were removed and counted using cell-counting methods described previously.

### 2.5. Cytokine Determination

To determine the cytokine levels a quantitative sandwich Enzyme-Linked Immuno Sorbent Assay (ELISA) (Cat. number KHG0111; Invitrogen) was performed per manufacturer's instructions. The conditioned media was then used to quantify VEGF levels according to manufacturer's instructions. Absorbances were measured at 450 nm using a Dynex Technologies MRXII plate reader equipped with Revelation 4.25 software. Concentrations of VEGF were quantified by comparing absorbances to their respective standard curves.

### 2.6. Statistical Methods

Data were analyzed using one-way analysis of variance (ANOVA). A *P*  value ≤ 0.05 is considered to be statistically significant and are indicated with “*”.

## 3. Results

### 3.1. Rb1-Induced ARPE-19 Cell Proliferation: Time and Dose Dependency

**Figure 1:**
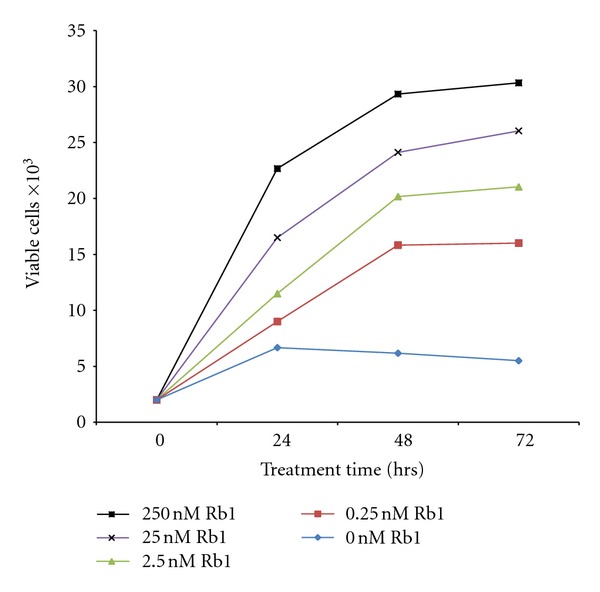
Effect of different concentrations of Rb1 on cell viability with increasing time. Cells were seeded at 20,000 per well on Day 0. The cells were treated with 0, 0.25, 2.5, 25, and 250 nM concentrations of Rb1, respectively. Cells were collected at 24, 48, and 72 hrs. Viable cells were counted using methods described previously. Media was unchanged within the 72 hr experimental period. Results indicate mean viable cell counts ± SE (*n* = 3).

A 3-day-dose dependency study show there is a significant increase in viable ARPE-19 cells in response to increased concentration of Rb1 and incubation time. Treatment with 0, 0.25, 2.5, 25, and 250 nM Rb1 resulted in cell proliferation in a dose- and time-dependent manner (Figure 1).

### 3.2. Rb1-Induced Downregulation of VEGF in ARPE-19 Cells in Culture

**Figure 2:**
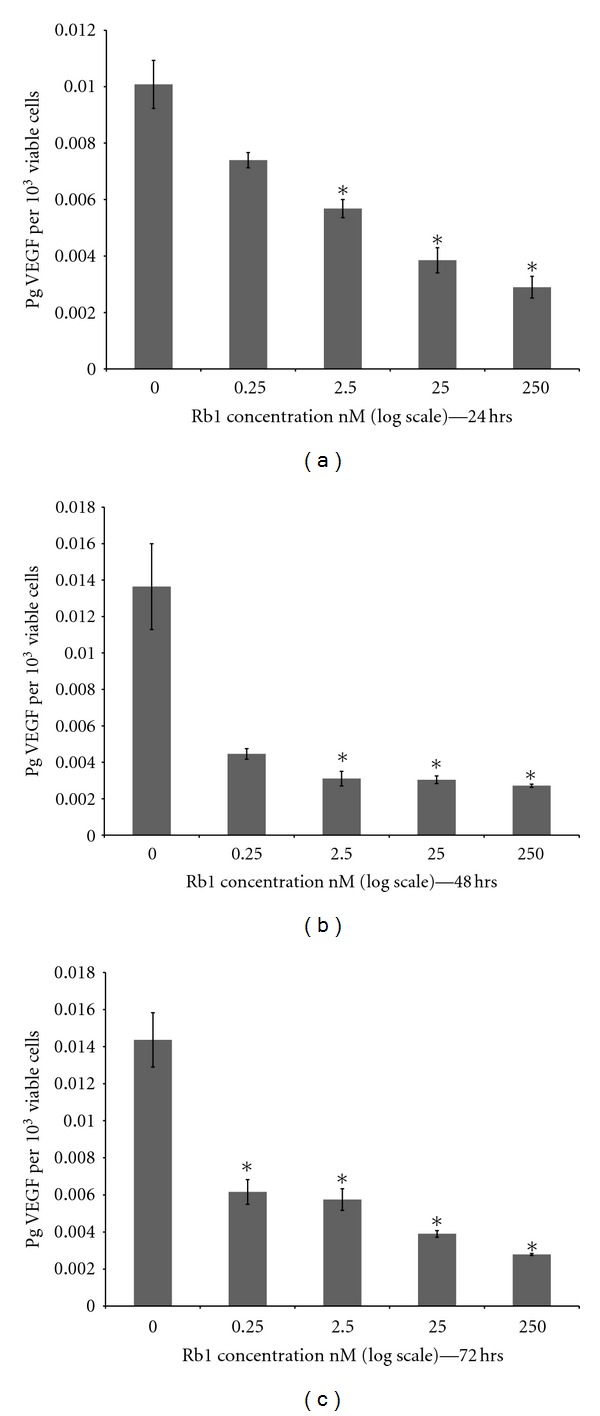
Effect of different concentrations of Rb1 on VEGF release with increasing time. Cells were seeded at 20,000 per well on day 0. The cells were treated with 0, 0.25, 2.5, 25, and 250 nM concentrations of Rb1 for 24, 48, and 72 hrs. Conditioned media was collected and ELISA performed for the total protein concentration for each day. The results shown in the graph indicate the mean pg of VEGF per 10,000 viable cells for the respective day ± SE. (a), (b), and (c) are the mean pg of VEGF per 10,000 viable cells when treated with different concentrations of Rb1 during the 0 hr to 72 hr treatment periods ± SE. Asterisks indicate statistical significance of *P* ≤ 0.05  (*n* = 3).

Treatment of ARPE-19 cells with increasing concentration of Rb1 (0, 0.25, 2.5, 25, and 250 nM) for 24, 48, and 72 hrs resulted in a significant decrease of VEGF (pg/10,000 viable cells) in the conditioned media (Figure 2(a)). Level of VEGF in the conditioned media collected at 24 hrs decreased in proportion to increasing (log) Rb1 concentration. Levels of VEGF in conditioned media collected in 48 and 72 hrs were significantly lower (30.77%; *P* ≤ 0.05) when treated with Rb1 (0.25, 2.5, 25, and 250 nM) when they are compared to VEGF levels in conditioned media without Rb1 (0.0 nM; Figures 2(b) and 2(c)).

### 3.3. Effect of Exogenous VEGF on Cell Viability

**Figure 3:**
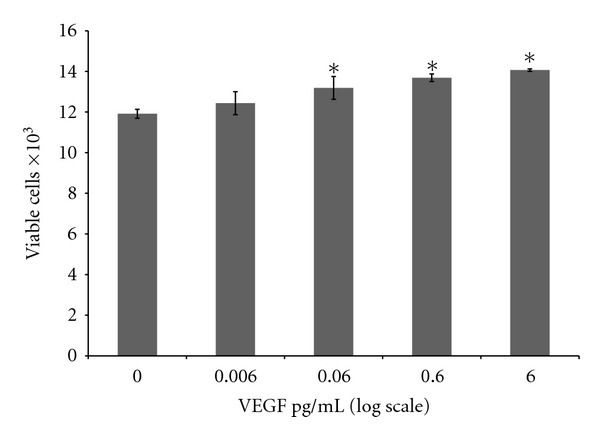
Effect of exogenous VEGF on cell viability. Cells were seeded at 20,000 per well on Day 0. The cells were treated with 0, 0.006, 0.06, 0.6, and 6 pg/mL concentrations of VEGF for 24 hrs. Viable cells were counted using methods described previously. Results indicate mean viable cell counts ± SE. Asterisks indicate statistical significance of *P* ≤ 0.05  (*n* = 3).

When ARPE-19 cells are treated with 0.006, 0.06, 0.6, and 6 pg/mL of exogenous VEFG, there is a small increase in viable cells (Figure 3) with significance only at 0.06, 0.6, and 6 pg/mL of VEFG.

### 3.4. Effect of Exogenous VEGF and Rb1 Cotreatment on Cell Viability

**Figure 4:**
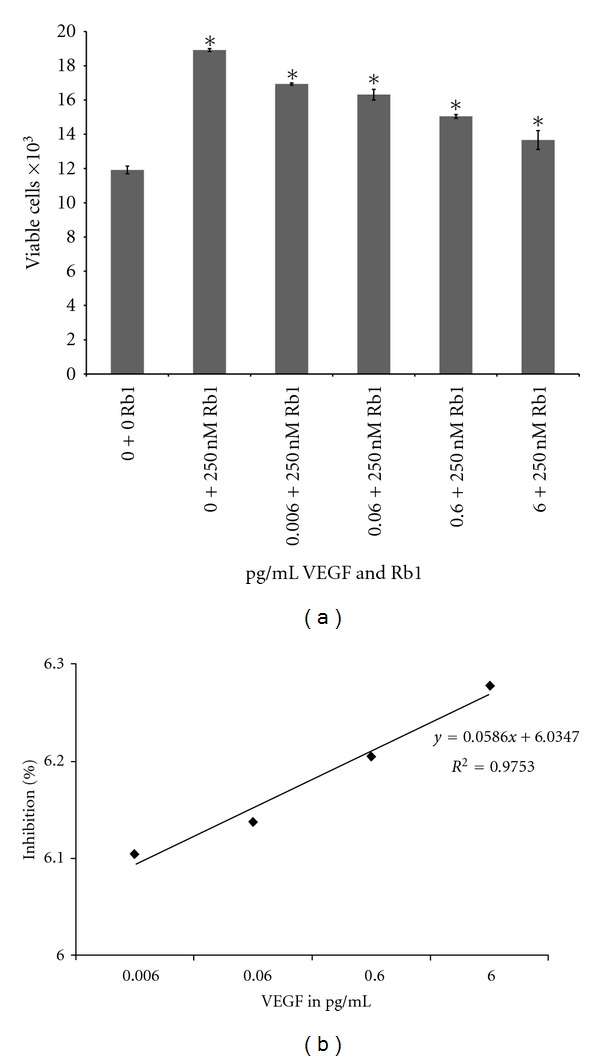
Effect of cotreatment of exogenous VEGF and 250 nM Rb1 on cell viability. (a) Cells were seeded at 20,000 per well on Day 0. Cells were co-treated with 250 nM Rb1 and 0, 0.006, 0.06, 0.6 and 6 pg/mL of VEGF respectively. Cells were incubated for 24 hrs and viable cell numbers were determined using methods described previously. Results indicate mean viable cell counts ± SE. Asterisks indicate statistical significance of *P* ≤ 0.05  (*n* = 3). (b) % Inhibition of cell viability due to the cotreatment (semi-log plot). The cell count data was compared when the cells were treated with 250 nM Rb1 versus cotreatment with Rb1 and different concentrations of VEGF for 24 hrs. The results indicate the percentage of inhibition with the cotreatment. The % inhibition is calculated by [viable cells with Rb1 treatment − viable cells with VEGF & Rb1 cotreatment]/[viable cells with Rb1 treatment − viable cells with no treatment (control)] using results shown in (a).

Treatment of ARPE-19 cells with 250 nM Rb1 for 24 hrs resulted in a significant increase in the number of viable cells by 58.8%  (*P* ≤ 0.01) when compared to 0.0 nM Rb1 (Figure 4(a)). However, cotreatment with VEGF resulted in significant decrease in viable cells (10.5%) when co-treated with 0.006 ng/mL VEGF, decrease by 13.7% (*P* ≤ 0.05) when treated with 0.06 ng/mL VEGF, decrease by 20.5% (*P* ≤ 0.01) when treated with 0.6 ng/mL VEGF and 27.8% (*P* ≤ 0.01) when treated with 6 ng/mL VEGF, respectively, compared to 250 nM Rb1; (Figure 4(a)). This significant inhibition of Rb1-induced cell proliferation was dose dependent (Figure 4(b)).

## 4. Discussion

Although previous studies have shown that ginsenosides possess a potent antiangiogenic effect, its action on ocular cells has not been studied. Results from the present study are the first to show that Rb1, a principal active ingredient of ginseng, significantly induced human RPE cell proliferation in a time- and dose-dependent manner (Figure 1). Moreover, treatment of ARPE-19 cells with Rb1 at different concentrations for 24, 48, and 72 hrs resulted in a significant reduction in VEGF (a known proangiogenic cytokine) levels (pg/10,000 viable cells) in the conditioned media (Figure 2), which may be a direct result of downregulation of its cellular synthesis and release. As RPE cells are located adjacent to choroidal capillaries and other major ocular vasculatures, and are the major contributor of VEGF in the eye, these finding suggests that Rb1 may have a strong effect on the inhibition of angiogenesis of choroidal and retinal capillaries, which contribute to the development of AMD and diabetic retinopathy.

Figure 3 shows that VEGF (up to 6 pg/mL for 24 hrs) resulted in a small increase in the number of viable cell. Figure 2(a) shows that Rb1 (at 250 nM for 24 hrs) resulted in a significant decrease in VEGF synthesis and release (per 10,000 cells). When co-treated with VEGF (6 pg/mL) and Rb1 (250 nM) for 24 hrs, the effect of Rb1 on the increase in cell number (see Figure 1), as a result of significantly a downregulated VEGF synthesis and release (per 10,000 viable cells; Figure 2(a)), far out-weighed the effects of exogenously added VEGF (6 pg/mL; Figure 3) on the small increase in cell number. Together with results that Rb1 treatment-induced cell proliferation and decreased VEGF release (Figures 1 and 2), results from ARPE-19 cells co-treated with VEGF and Rb1 (Figure 4) strongly suggest that Rb1's effect on the increase in RPE cells may be a direct result of its significant inhibition on VEGF synthesis and release by these cells. 

Currently, there are few pharmacokinetic studies on the effects of ginsenosides in humans [22]. Because there is limited literature discussing plasma concentration of ginsenosides in humans, the physiological or toxic levels of ginsenoside Rb1 in human blood is unclear [23–25]. Sengupta et al. studied ginsenoside Rb1's angiogenesis effects at the concentrations of 0.125, 1.25, and 125 nM, respectively. Leung and associates used 100, 150, 250 and 500 nM concentrations of Rb1 in HUVEC's and observed a significant decrease in tube formation following treatment with 250 nM Rb1. Therefore, we selected 250 nM of Rb1 for our experiments.

The particular pathways involved in how this Rb1 mediates its effects should be further studied. As ginsenosides are steroidal compounds, which are structurally similar to estrogen, it is possible that Rb1 binds to estrogen receptor to affect a downstream pathway. Recent investigations from our laboratory indicate that with the increasing concentration of estrogen there is a decrease in VEGF and PEDF in Rhesus monkey retinal capillary endothelial cells [26]. Further studies will be needed to investigate how Rb1 exert its effect on RPE cells via estrogen receptors [3].

Retinal angiogenesis is an extensively studied, yet not fully understood process in which retinal vascular endothelial cells proliferate and migrate through a damaged vessel basement membrane and form tubules that can circulate blood [27–29] and is signaled by VEGF secretion. This neovascularization leads to impaired vision. Based on results from the present study, the ginsenoside Rb1 might be considered as an important homeopathic or pharmacological treatment for ocular diseases like AMD which involves the process of choroidal neovascularization mediated by the potent angiogenic growth factors VEGF.

## 5. Conclusions

In summary, we report a significant effect of Rb1 on the viability of ARPE-19 cells and found that Rb1 significantly decreased cellular VEGF production, a pro-angiogenic cytokine in ARPE-19. This is the first report of antiangiogenic function of Rb1 in an important ocular cell type.

Understanding the correlation between the growth factors and their dependence on specific cell types is essential for future pharmacological studies. Rb1 is a potential antiangiogenic agent which may decrease choroidal neovascularization, thus providing a new method of intervention of AMD and other related types of angiogenic ocular diseases.
